# Caregiver water safety knowledge and views of toddler water competency

**DOI:** 10.1186/s40621-023-00479-w

**Published:** 2023-12-13

**Authors:** Molly B. Johnson, Karla A. Lawson

**Affiliations:** 1 Trauma and Injury Research Center, Dell Children’s Medical Center, 4900 Mueller Blvd, Austin, TX 78723 USA; 2 Kinesiology Department, University of the Incarnate Word, San Antonio, TX 78209 USA; 3 Department of Surgery and Perioperative Care, Dell Medical School, University of Texas at Austin, Austin, TX 78712 USA

**Keywords:** Swimming, Water competency, Drowning, Supervision, Parents, Toddlers, Pediatric, Submersion

## Abstract

**Background:**

Drowning is the leading cause of death for toddlers. When caregivers are knowledgeable about water safety, they can provide the best protection against drowning. The aim of this study is to survey caregivers of toddlers to better understand factors associated with water safety knowledge, attitudes about pool supervision, and toddler water competency skills.

**Methods:**

An online survey of 650 parents/caregivers of 1–4-year-old toddlers asked about the caregiver’s water safety and swimming background and views on pool supervision. Surveys included a true/false section of ten basic water safety knowledge questions. Caregivers also reported on toddler swim lesson history and whether their toddler could perform six standard water competency skills. Linear regression identified factors predictive of water safety knowledge.

**Results:**

On average, caregivers selected the correct answer on six out of ten water safety knowledge questions. Water safety knowledge was predicted by the relationship of the caregiver to the toddler, gender, race, education, prior CPR training, caregiver swim capability, and reported pool supervision style. On average, caregivers reported that their toddler could perform half of the water competency skills. The majority of the toddlers had taken swimming lessons. One third of caregivers believed that after a toddler has had swimming lessons, they don’t need to be watched as closely when they are in a pool.

**Conclusions:**

Findings suggest that water safety knowledge is poor and that there are misconceptions about toddler supervision needs. Efforts are needed to improve water safety knowledge and to change perceptions about supervision among caregivers of toddlers.

## Background

Within the USA, more toddlers die from drowning than from any other cause (National Center for Injury Prevention and Control 2022). Furthermore, nonfatal drowning is a significant cause of injury, with seven children treated for nonfatal submersion injury for every one fatal drowning (National Center for Injury Prevention and Control 2022). Submersion injuries have a high morbidity rate, with almost 40% of submersion injuries requiring hospital admission compared with 10% for other accidental injury mechanisms (National Center for Injury Prevention and Control 2022). It is critical we understand factors that are associated with drowning risk so we can work to prevent drowning fatalities and injuries.

Recommendations by the American Academy of Pediatrics highlight the need for multiple layers of protection against drowning. Layers of protection include the use of home pool security features, swimming where lifeguards are present, wearing United States Coast Guard approved life jackets, training in CPR, developing children’s water competency, and supervising children when they are in or near a body of water (Denny et al. 2021). Without multiple layers of protection, children may be at risk of drowning. However, if caregivers do not know what layers of protection are important, they may not put them into practice.

For parents and caregivers in the USA, it is unclear what the typical level of understanding of drowning prevention and water safety knowledge is. Research suggests that risky misconceptions about water safety are common (Mackay et al. 2016). Additionally, it is not known what demographic and background factors influence water safety knowledge. The first aim of this study is to assess water safety knowledge of caregivers of toddlers using a set of basic true/false questions and to identify predictors of water safety knowledge.

Research highlights how lapses in supervision play a critical role in drowning incidents (Quan and Cummings 2003). Yet caregiver’s of toddlers report a willingness to reduce supervision of toddlers around water to perform mundane tasks like preparing food or texting (Johnson et al. 2021). Additionally, supervision of toddlers around swimming pools is predicted by the caregivers’ perceptions of supervision needs (Johnson et al. 2021). Misconceptions, then, could lead caregivers to make poor supervision choices. For example, parents may believe that their toddler needs less close and attentive supervision after a course of swimming lessons (Morrongiello et al. 2014). Parents may overestimate a child’s swimming ability (Morrongiello et al. 2014). Additionally, parents may have the misconception that children can keep themselves safe from drowning (Morrongiello et al. 2013). Research does show that toddlers who participated in swimming lessons had a reduced risk of drowning (Brenner et al. 2009), but even toddlers with the strongest water competency skills are still at risk. The second aim of this study is to understand views about the role of swim lesson experience on swimming pool supervision.

With caregivers potentially basing supervision decisions around perceived water competency of their toddler, a better understanding of caregiver estimates of the water competency skill level of their toddlers is needed. The third aim of this study is to explore toddler water competency skills and swim lesson experience reported by their caregivers.

## Methods

### Survey procedures

We conducted a cross-sectional study using an anonymous survey of caregivers of toddlers. We recruited participants using the online Amazon MTurk platform. MTurk is a common academic resource that offers access to a large participant pool, has been found to be representative of the general USA population, and is a source of quality research data (McCredie and Morey 2019). Only MTurk workers who lived in the USA and had a 95% or higher approval rating on prior MTurk projects were given access to the recruitment information. MTurk workers could only begin the survey if they answered that they were the caregiver for a toddler (1–4 years old) and 18 years of age or older. Participants reviewed and agreed to an informed consent document before starting the survey. Participants were paid $2 for completing the survey. The study was approved by the University of Texas at Austin Health Sciences Institutional Review Board.

### Survey instrument

**Fig. 1:**
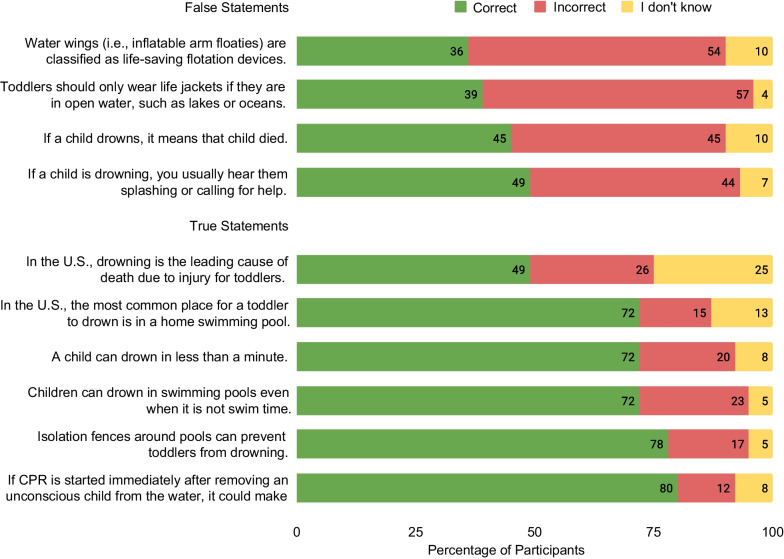
Participant answer selections on true/false water safety knowledge statements

The survey asked about the demographics and background of the participant and the toddler they cared for. The water safety knowledge section of the survey consisted of ten questions designed to test basic knowledge related to drowning and water safety that might be useful for the caregiver of a toddler (Fig. 1). For each question, they could answer true, false, or I don’t know about each knowledge statement. We created a total knowledge score by summing all correct answers. There was a possible range of scores from 0 to 10, with 0 reflecting low knowledge and 10 reflecting high knowledge. The water competency skills section asked caregivers whether their toddler was able to perform the following six standard water competency skills: (1) Enter water above his/her head and return to the surface, (2) turn around and then find an exit from the pool, (3) float unassisted for 1 min, (4) tread water for 1 min, (5) exit the pool without using a ladder, and (6) swim 25 m without stopping (80 feet, the length of a standard pool). For each skill, participants could choose Yes, No, or I don’t know.

We assessed swimming pool supervision style by asking, “When your toddler is in a pool, what do you most often do?” We assessed swim frequency with the question, “During the summer, about how often do you swim with your toddler? (Please answer the way you would if there were no restrictions due to the coronavirus.)” We assessed caregivers swimming capability by asking, “Can you swim 25 m (80 feet, the length of a standard pool) without touching the bottom?” We asked whether the caregiver had taken CPR training and whether their toddler had taken swimming lessons. We also asked caregivers to rate their agreement with the statement, “After a toddler has had swimming lessons, they don’t need to be watched as closely when they are in the pool.” The survey questions were developed based on a review of water safety literature (Mackay et al. 2016; Morrongiello et al. 2014, 2013; Glassman et al. 2018; Sandomierski et al. 2019). The survey was tested on caregivers of toddlers during development. Face validity was assessed through reviews by three experts in the field.

### Data analysis

The inclusion of quality control items is recommended when collecting data using Mturk (Agley et al. 2022). To ensure the quality of our data, we scored a number of data quality indicators, such as contradictory answers on multiple choice questions. Of 916 completed surveys, we excluded 266 surveys with more than 2 low-quality indicators from analyses. We performed scoring of water safety knowledge and water competency skills sections in Excel. We conducted all statistical analyses using STATA (version 12.1, STATA, inc, College Station, TX). We analyzed ten demographic variables as possible predictors of water safety knowledge. We included variables with significant differences in knowledge scores at *P* < .05 on univariate analyses (Table 1) in a multivariate linear regression model with the water safety knowledge score as the dependent variable. To compare demographic groups, we present coefficients from the regression results as percentage differences in scores (e.g., a coefficient of 1.2, which represents a score of 1.2 above the reference group score is presented as a 12% higher knowledge score). We used analysis of variance to assess whether there were differences in the number of water competency skills reported by toddler age, with post hoc Bonferroni pairwise comparisons made between age groups.

**Table 1:** Characteristics of survey participants and mean water safety knowledge scores

Category	*n*	%	Mean water safety score	Standard deviation	*P* value
Caregiver relationship					
Parent	520	80.0	6.41	2.14	.0002
Grandparent	58	8.9	6.14	1.97	
Aunt/uncle/cousin	44	6.8	6.39	2.39	
Sibling	28	4.3	4.57	2.13	
Gender					
Female	248	38.2	6.75	2.25	< .0001
Male	402	61.8	6.03	2.07	
Age (years)					
18–24	23	3.5	6.09	2.00	.0016
25–34	352	54.2	6.13	2.15	
35–44	200	30.8	6.82	2.21	
45–54	47	7.2	5.72	1.97	
55 or older	28	4.3	6.11	2.01	
Race/ethnicity					
White	461	70.9	6.41	2.21	< .0001
Black	118	18.2	5.48	1.77	
Asian	18	2.8	7.67	1.75	
Multiple races	22	3.4	6.59	2.28	
Hispanic/Latino	31	4.8	6.84	2.18	
Income					
Less than $25,000	44	6.8	6.61	2.05	.0005
$25,000–$49,999	197	30.3	6.06	2.11	
$50,000–$99,999	330	50.8	6.20	2.21	
$100,000 or more	79	12.2	7.21	2.00	
Education					
High school or less	40	6.2	7.35	2.34	< .0001
Some college	84	12.9	7.39	2.12	
Bachelor’s degree	409	62.9	6.00	2.14	
Advanced degree	117	18.0	6.25	1.90	
All caregivers	650	100	6.31	2.17	

## Results

We analyzed results for 650 participants. A majority of the participants were parents (parents/foster parents/step-parents) of a toddler (80%), male (62%), white (71%), 25–34 years old (54%), with a bachelor’s degree (63%), and had an income of $50,000–$99,999 (51%) (Table 1). There was at least one participant from every state in the USA except South Dakota and Alaska. Many caregivers had prior CPR training (71%), could easily swim 25 m (61%), reported an arm’s reach supervision style (67%), and swam with their toddler a few times a summer (40%) (Table 2). The majority of the toddlers cared for were male (67%).

**Table 2:** Survey participant background and mean water safety knowledge scores

Category	*n*	%	Mean water safety score	Standard deviation	*P* value
CPR training					
No CPR training	190	29.2	5.83	2.23	.0003
Prior CPR training	460	70.8	6.50	2.11	
Swim capability					
Definitely not	19	2.9	7.53	1.87	< .0001
Probably not	28	4.3	5.79	2.18	
Yes, but it would be hard	208	32.0	5.44	1.99	
Yes, easily	395	60.8	6.74	2.12	
Supervision style					
Listen from a distance and look over to check on them occasionally	15	2.3	4.67	1.50	< .0001
Stay nearby and look at them frequently (e.g., on the side of the pool)	203	33.6	5.41	2.06	
Stay within arm’s reach and watch them constantly	432	66.5	6.78	2.08	
Swim frequency					
Never	45	6.9	6.00	2.37	.6398
A few times a summer	258	39.7	6.27	2.10	
A few times a month	177	27.2	6.38	2.20	
A few times a week	115	17.7	6.49	2.14	
Most days	55	8.5	6.09	2.30	

### Water safety knowledge

On average, caregivers selected the correct response on 6.31 (SD = 2.17) out of 10 true/false questions, with a range of scores of 0–10. Only 6% of participants responded correctly to all 10 knowledge questions. More than one third of participants did not give the correct answer to the majority of the true/false questions. Participants were most knowledgeable about the usefulness of CPR as a first response for drowning and least knowledgeable about flotation devices (Fig. 1). Fewer than half of participants knew that you do not usually hear splashing or calling for help when someone is drowning or that drowning is the leading cause of death due to injury for toddlers.

### Predictors of water safety knowledge

Univariate analyses showed a significant association with water safety knowledge for nine variables, which were included in a multivariate regression model (Table 1, 2, 3). The multivariate linear regression model explained 27% of the variance in water safety knowledge (*F*(24, 625) = 9.71, *P* < .001. *R*^2^ = 0.2715). Seven variables were found to be significant predictors of water safety knowledge: caregiver relationship, gender, race/ethnicity, education, CPR training, swim capability, and supervision style.

**Table 3:** Linear regression model results for water safety knowledge score

Category	Coefficient	Standard error	*P* value	95% Confidence interval	
Caregiver relationship					
Parent					
Aunt/uncle/cousin	− 0.02	0.30	0.94	− 0.61	0.56
Grandparent	− 0.12	0.29	0.67	− 0.69	0.45
Sibling	− 1.20	0.38	0.00	− 1.95	− 0.45
Gender					
Female					
Male	− 0.49	0.16	0.00	− 0.80	− 0.18
Age (years)					
18–24					
25–34	− 0.02	0.42	0.97	− 0.83	0.80
35–44	0.32	0.43	0.46	− 0.52	1.15
45–54	− 0.29	0.50	0.56	− 1.26	0.68
55 or older	− 0.01	0.56	0.99	− 1.10	1.09
Race/ethnicity					
White					
Black	− 0.60	0.20	0.00	− 1.00	− 0.21
Asian	0.72	0.47	0.12	− 0.19	1.64
Multiple races	0.11	0.42	0.80	− 0.71	0.17
Hispanic/Latino	0.17	0.36	0.64	− 0.53	0.87
Income					
Less than $25,000					
$25,000–$49,999	− 0.21	0.32	0.52	− 0.84	0.43
$50,000–$99,999	− 0.01	0.32	0.97	− 0.63	0.61
$100,000 or more	0.64	0.37	0.09	− 0.10	1.37
Education					
High school or less					
Some college	− 0.07	0.37	0.85	− 0.79	0.65
Bachelor’s degree	− 1.11	0.33	0.00	− 1.75	− 0.47
Advanced degree	− 0.96	0.36	0.01	− 1.67	− 0.26
CPR					
No CPR training					
Prior CPR training	0.65	0.17	0.00	0.31	0.98
Swim capability					
Definitely not					
Probably not	− 1.16	0.57	0.04	− 2.28	− 0.03
Yes, but it would be hard	− 1.49	0.47	0.00	− 2.42	− 0.57
Yes, easily	− 0.75	0.46	0.11	− 1.65	0.16
Supervision style					
Listen from a distance and look over to check on them occasionally					
Stay nearby and look at them frequently (e.g., on the side of the pool)	0.49	0.51	0.34	− 0.51	1.50
Stay within arm’s reach and watch them constantly	1.31	0.51	0.01	0.32	2.31

According to the coefficients presented in the regression results in Table 3, some groups showed higher water safety knowledge scores, on average, than others. Parents scored 12% higher than siblings. Female caregivers scored 5% higher than male caregivers and White caregivers scored 6% points higher than Black caregivers. Caregivers with a high school education scored 10–11% higher than those with a bachelor's degree or advanced degree. Caregivers who had taken a CPR class scored 7% higher than those who had not. Swim capability results showed that non-swimmers who could “definitely not” swim 25 m scored 12–15% higher than poor swimmers who answered “probably not” or “yes, but it would be hard,” but were not significantly different from those who could “easily” swim 25 m. Caregivers who reported their supervision style as “stay within arm’s reach and watch them constantly” while in a swimming pool scored 13% higher than caregivers whose supervision style was “listen from a distance and look over to check on them occasionally.”

### Water competency skills

On average, caregivers reported that their toddler could perform half of the six water competency skills (mean 2.85; SD: 1.95), with answers ranging between the minimum possible number (0) and the maximum possible number (6). The number of water competency skills reported by the caregiver differed depending on the age of the toddler (*F*(3,646) = 11.65, *P* < .001). One-year-olds were reported to have significantly fewer water competency skills than older age groups and four-year-olds to have significantly more water competency skills than younger age groups (Table 4). Two- and three-year-olds were not significantly different from each other.

**Table 4:** Mean number of water competency skills by toddler age

Toddler age	*n*	%	Mean water competency skills	Standard deviation
1 year	57	8.8	1.88^a^	2.22
2 years	159	24.5	2.68^b^	1.95
3 years	233	35.9	2.75^b^	1.96
4 years	201	30.9	3.41^c^	1.95

Mean number is out of six possible water competency skills; means not followed by the same superscript letter were significantly different from each other in Bonferroni pairwise comparisons

In all age groups, fewer toddlers were reported to be able to swim 25 m without stopping or exit the pool without using a ladder than other skills, with about one third of toddlers reportedly able to perform these lifesaving skills. See Table 5 for percentages of toddlers reported to perform each skill.

**Table 5:** Percentage of toddlers within each age group that can perform each skill

Water competency skill	1 year	2 years	3 years	4 years	All ages
Enter water above his/her head and return to the surface	36.8	62.9	58.4	75.6	62.9
Turn around and then find an exit from the pool	35.1	52.20	54.9	63.7	55.2
Float unassisted for 1 min	29.8	49.7	51.5	60.7	52.0
Tread water for 1 min	26.3	42.1	43.8	61.2	47.2
Exit the pool without using a ladder	28.1	36.5	32.2	40.8	35.5
Swim 25 m without stopping (80 feet, the length of a standard pool)	31.6	31.5	27.0	39.3	32.3
None of the six skills	49.1	21.4	21.0	7.5	19.4
All six skills	10.5	8.8	9.9	11.0	10.0

### Swimming lessons

Fifty-nine percent of caregivers reported that their toddler had taken swimming lessons. Thirty percent of caregivers planned for their toddler to take swimming lessons. Eleven percent of caregivers did not plan for their toddler to take swimming lessons.

### Swimming pool supervision

Attitudes about whether supervision can be loosened after toddlers have swimming lessons were divided. Of all the caregivers surveyed, 36% agreed or strongly agreed, 47% disagreed or strongly disagreed, and 18% were neutral about the statement, “After a toddler has had swimming lessons, they don’t need to be watched as closely when they are in a pool.”

## Discussion

This study highlights factors associated with caregiver water safety knowledge. The relationship of the caregiver to the toddler is a factor, with parents more knowledgeable than siblings, but not grandparents or aunts/uncles. Results suggest that there are knowledge gaps for certain groups, particularly male and Black caregivers and those who can swim, but poorly. Education showed a relationship opposite what might be expected, with lower education levels associated with higher water safety knowledge. In contrast, education specific to lifesaving, from prior CPR training, was associated with higher water safety knowledge. Additionally, higher water safety knowledge was predicted by caregiver reports of closer and more attentive supervision of their toddler around water.

Caregivers reported that the majority of toddlers had taken swimming lessons. Caregivers thought their toddler could perform an average of three out of six water competency skills. Additionally, survey results highlighted a common misconception about supervision: more than one third of caregivers believed that toddlers did not need to be watched as closely in a swimming pool after they had swimming lessons.

Overall, this study found that water safety knowledge was not high among caregivers of toddlers. Our results for 49% of caregivers match prior findings that 48% of parents in the USA falsely believed they would hear a child if they were drowning (Mackay et al. 2016). Considering that drowning is the leading cause of death for toddlers (National Center for Injury Prevention and Control 2022), it is clear that efforts need to be made to improve caregivers’ understanding of water safety. Caregivers lack critical knowledge, such as how to identify someone who is drowning or what flotation devices are best. It is unclear what types of water safety programming will most effectively lead caregivers to make safer supervision decisions around water. Research supports the effectiveness of a wide range of water safety education programming types to improve knowledge (Glassman et al. 2018; Sandomierski et al. 2019; Matthews and Franklin 2018; Denehy et al. 2017). Our data showed that people who had taken CPR classes were more knowledgeable about water safety. However, it is unclear if their greater knowledge came from the CPR training or whether these people were generally more motivated to educate themselves about water safety, including CPR. More research is needed to understand barriers to accessing and learning water safety and drowning prevention information and implementing supervision behavior changes.

Certain demographic groups may be more in need of water safety knowledge than other groups. Our data suggest that male and Black caregivers know less about water safety. Across all age groups, these same demographic groups are most at risk of drowning (Mackay et al. 2016; Johnson et al. 2022a; Moreland et al. 2022). Clearly, water safety initiatives need to target these high-risk groups for their own safety as well as for the safety of the children they care for.

Additionally, adults who are poor swimmers may have a greater need for general water safety information, but also for water competency/swimming instruction themselves. With arms reach supervision recommended when supervising toddlers in a swimming pool (Denny et al. 2021), it is essential the caregiver is comfortable and capable enough in the water to provide that supervision. Our prior research suggests that swimming capability and perceptions of swimming pool supervision of toddlers are intertwined, with poor swimmers viewing arm’s reach supervision as less necessary than good swimmers or non-swimmers (Johnson et al. 2022b). Adults lacking enough personal experience with water could have inadequate awareness of the dangers it poses to children in their care.

Supervision perceptions and behaviors could be guided by false information, not simply an absence of information. Prior research has shown that parents believe swimming lessons may be drown-proofing children (Morrongiello et al. 2013). Even though swimming lessons may slightly reduce the chances of a child drowning (Brenner et al. 2009), multiple layers of protection must be in place to truly prevent drowning (Denny et al. 2021). This study reveals that many caregivers believe pool supervision can be reduced after a toddler has had swimming lessons. This type of misconception about swimming and drowning may be shaping supervision around water. Education is needed to dispel myths that can have hazardous consequences.

As might be expected, caregivers reported a higher number of water competency skills for four-year-olds and a lower number of skills reported for one-year-olds. However, research suggests that self-reported and actual swimming capability are not always matched; in fact, caregivers may overestimate the water competency of children (Morrongiello et al. 2014). Our data show that one third of 1-year-old toddlers were reported to be able to swim 25 m. Even though this age group includes toddlers who are almost 2 years old, it is possible this represents some overestimation of actual water competency. Such overconfidence in toddlers’ swim skills could lead caregivers to make poor supervision choices.

There are limitations to this study. Although a number of demographic characteristics were predictive of water safety knowledge, the model in this study only accounted for 27% of the variance in knowledge. We were limited to analyzing only self-report information from the survey, so it is unclear what additional factors might be important. Potentially prior experience matters. Research should assess whether having older children or knowing someone who has drowned could motivate someone to gain a better understanding of drowning prevention and water safety. Additionally, because our sample included a majority of male participants and included caregiving relatives besides parents, results might be different than a more representative sample of parents. Since caregiver status was self-selected, we do not know how much regular responsibility that person actually had for the toddler they reported on. They may have had more limited knowledge of their water competency or swim lesson attendance than a survey of only parents. Some of our results are unclear as well, for example, we have no explanation for why a lower education level was associated with higher water safety knowledge.

Prior research shows that perceptions of arm’s reach pool supervision and water safety knowledge are predictive of self-reported supervision behavior of the caregiver with their own toddler (Johnson et al. 2021). However, self-reported perceptions and behaviors may not be the same as actual behavior adopted by caregivers when they are supervising toddlers around water. Observational research is needed to assess actual supervision behavior in various water settings.

## Conclusions

This study revealed that caregivers of toddlers have a low level of water safety knowledge and that knowledge varies based on demographic and background factors. This study supported a link between water safety knowledge and views about supervision. Additionally, it highlighted misconceptions about supervision needs of toddlers after taking swimming lessons. Future research is needed to better understand actual supervision behavior of toddlers around water and to identify the most effective approaches to improve water safety knowledge and dispel myths that could mislead parents and caregivers into making risky choices around water.

## Data Availability

We do not have the consent of the participants to make data public.

## Abbreviations

CPR: Cardiopulmonary Resuscitation
MTurk: Amazon Mechanical Turk
Parents: Parents/foster parents/step-parents
SD: Standard deviation
USA: United States of America
